# Initial Intraoperative Experience with Robotic-Assisted Pedicle Screw Placement with Cirq^®^ Robotic Alignment: An Evaluation of the First 70 Screws

**DOI:** 10.3390/jcm10245725

**Published:** 2021-12-07

**Authors:** Mirza Pojskić, Miriam Bopp, Christopher Nimsky, Barbara Carl, Benjamin Saβ

**Affiliations:** 1 Department of Neurosurgery, University of Marburg, 65199 Marburg, Germany; bauermi@med.uni-marburg.de (M.B.); nimsky@med.uni-marburg.de (C.N.); Barbara.carl@helios-gesundheit.de (B.C.); sassb@med.uni-marburg.de (B.S.); 2 Marburg Center for Mind, Brain and Behavior (MCMBB), 65199 Marburg, Germany; 3 Department of Neurosurgery, Helios Dr. Horst Schmidt Kliniken, 65199 Wiesbaden, Germany

**Keywords:** robotic-guided spine surgery, pedicle screw accuracy, screw trajectory, screw entry point, entry point deviation, screw tip deviation, screw angular deviation

## Abstract

Background: Robot-guided spine surgery is based on a preoperatively planned trajectory that is reproduced in the operating room by the robotic device. This study presents our initial experience with thoracolumbar pedicle screw placement using Brainlab’s Cirq^®^ surgeon-controlled robotic arm (BrainLab, Munich, Germany). Methods: All patients who underwent robotic-assisted implantation of pedicle screws in the thoracolumbar spine were included in the study. Our workflow, consisting of preoperative imagining, screw planning, intraoperative imaging with automatic registration, fusion of the preoperative and intraoperative imaging with a review of the preplanned screw trajectories, robotic-assisted insertion of K-wires, followed by a fluoroscopy-assisted insertion of pedicle screws and control iCT scan, is described. Results: A total of 12 patients (5 male and 7 females, mean age 67.4 years) underwent 13 surgeries using the Cirq^®^ Robotic Alignment Module for thoracolumbar pedicle screw implantation. Spondylodiscitis, metastases, osteoporotic fracture, and spinal canal stenosis were detected. A total of 70 screws were implanted. The mean time per screw was 08:27 ± 06:54 min. The mean time per screw for the first 7 surgeries (first 36 screws) was 16:03 ± 09:32 min and for the latter 6 surgeries (34 screws) the mean time per screw was 04:35 ± 02:11 min (*p* < 0.05). Mean entry point deviation was 1.9 ± 1.23 mm, mean deviation from the tip of the screw was 2.61 ± 1.6 mm and mean angular deviation was 3.5° ± 2°. For screw-placement accuracy we used the CT-based Gertzbein and Robbins System (GRS). Of the total screws, 65 screws were GRS A screws (92.85%), one screw was a GRS B screw, and two further screws were grade C. Two screws were D screws (2.85%) and underwent intraoperative revision. There were no perioperative deficits. Conclusion: Brainlab’s Cirq^®^ Robotic Alignment surgeon-controlled robotic arm is a safe and beneficial method for accurate thoracolumbar pedicle screw placement with high accuracy.

## 1. Introduction

There were approximately 3.6 million spine surgery cases in the United States between 2001 and 2010, with increasing prevalence each year [1]. Computer-aided navigation and robotic guidance systems have become widespread in their utilization for spine surgery [2]. The coupling of surgical navigation with intraoperative computerized tomography (iCT) has improved the positional accuracy of pedicle screw placement [3,4] and reduced the operating theater staff’s exposure to ionizing radiation [5,6].

With the development of medical imaging technology and computer technology, both image navigation and robot technology have been gradually applied to pedicle screw placement [7,8,9]. It has been reported that robot-assisted pedicle screw placement achieves higher accuracy, lower radiation exposure and fewer complications [10,11].

Guidance to the surgeon in robot-guided spine surgery is based on a preoperatively planned trajectory that is reproduced in the operating room by the robotic device. In one variant, this device is mounted to the patient’s spine for drilling holes in preparation for the placement of pedicle screws. The bone-mounting feature allows patient breathing and motion without altering the position of the robotic unit relative to the spine, which maintains the system’s accuracy [9,12].

Robot-assisted spine surgery, which can provide an efficient and accurate mode of hardware placement, is relatively new and still seldom used by spine surgeons [13]. As with any new surgical technology, there is a significant learning curve involved with the robotic system [13]. This study presents our initial experience with thoracolumbar pedicle screw placement using the Brainlab’s Cirq^®^ surgeon-controlled robotic arm (BrainLab, Munich, Germany). The robotic arm is mounted onto a standard radiolucent OR table, and the surgeon positions the arm, which, once roughly aligned above to entry point of the planned trajectory, automatically adapts to the planned trajectory, and provides a channel for drilling with a drill guide and the implantation of K-wires, followed by pedicle-screw placement. This is currently the only robotic system that provides automatic adaptation to the preplanned trajectory. To our knowledge, this is the first case series to evaluate the use of CIRQ^®^ Robotic Alignment (BrainLab, Munich, Germany) for pedicle screw placement.

## 2. Materials and Methods

Informed consent was obtained from all individual participants included in this observational study. We obtained ethics approval for the prospective archiving of clinical and technical data, applying intraoperative imaging and navigation (study no. 99/18).

All surgeries were performed by a team of two surgeons with three (M.P.) and five (B.S.) years of experience in complex spinal surgery. All patients who underwent robotic-assisted implantation of pedicle screws in the thoracolumbar spine were included in the study. A total of 70 pedicle screws were evaluated. 

### 2.1. Workflow

Our workflow included the following: preoperative imagining, screw planning, intraoperative imaging with automatic registration, fusion of the preoperative and intraoperative imaging with a review of the preplanned screw trajectories, robotic-assisted insertion of K-wires followed by fluoroscopy-assisted insertion of pedicle screws and a control iCT scan. 

**Figure 1:**
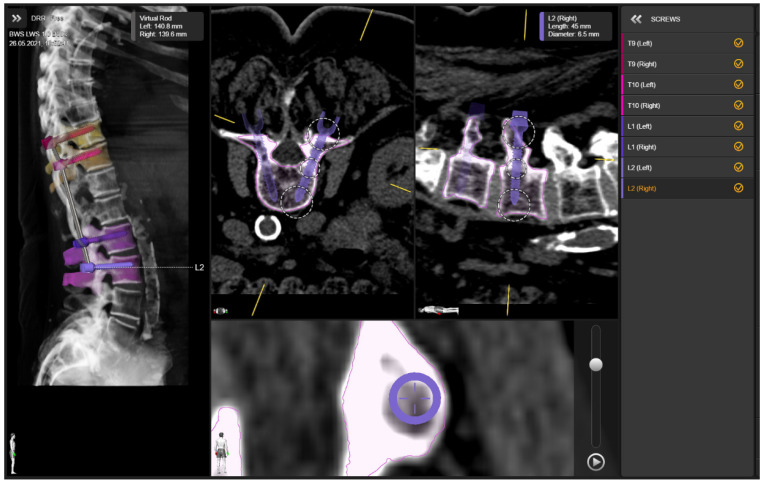
Preoperative screw planning using Screw planning application (BrainLab, Munich, Germany).

Prior to surgery, all patients received a 3D CT of the region of the spine that was planned for instrumentation. This dataset was exported to the navigation software (BrainLab, Munich, Germany). Using the crew planning application, the radius, length and trajectory of the pedicle screws were planned prior to surgery (Figure 1). The software automatically recognizes the vertebras which are planned for instrumentation and automatically sets the screws. In the next step, the screw direction, radius, and length were adjusted manually. After the trajectory was planned, a review of the trajectory was performed with final adoption. 

**Figure 2:**
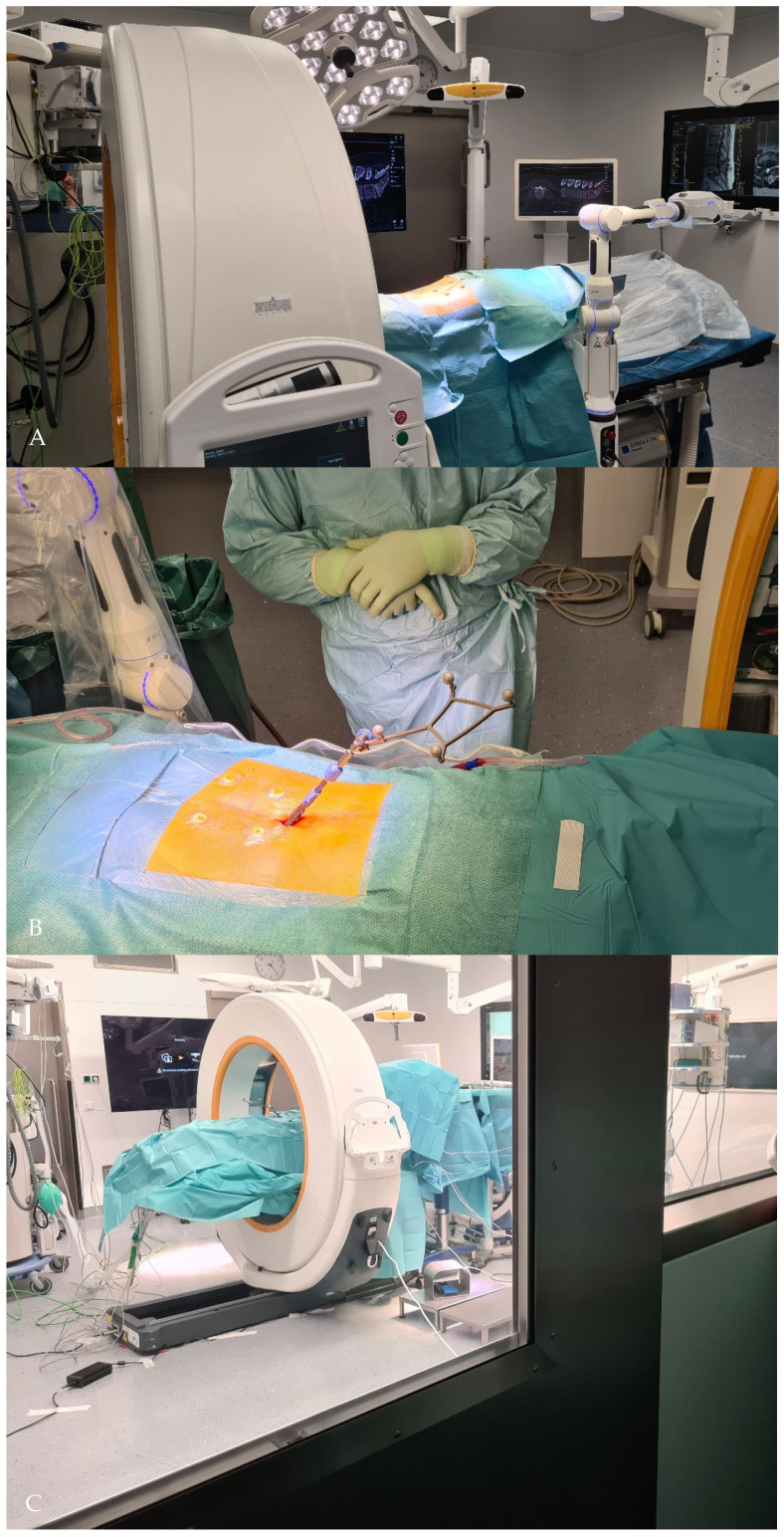
Perioperative setting. (**A**). Cirq^®^ is attached on the left side of the operating table. (**B**). Surgical field is prepped and draped. Four fiducials are attached to the skin prior for accuracy check. Reference array is in this case of percutaneous, minimally invasive pedicle screw implantation, is attached to a spinous process proximal to the surgical field via separate skin incision. (**C**). View of the patient while performing initial registration iCT scan.

The intraoperative setting with iCT has been described elsewhere [14]. The patient was placed on a mobile, radiolucent carbon-fiber surgical table (TruSystem^®^ 7500, Trumpf Medical Ditzinger, Germany) for spinal applications, connected to the CT scanner (AIRO^®^, Brainlab, Munich, Germany). Surgery was performed in the scanning position with anesthesia cables and lines routed through the gantry. Cirq^®^ was attached and draped on the left side of the operating table on a metal holder. (Figure 2.)

The correct spinal levels planned for the instrumentation were determined by fluoroscopy for planning the skin incision. Four artificial adhesive skin fiducials were placed on both sides of the planned incision to monitor accuracy. 

The surgical field was prepped and draped. A carbon reference array was attached to the spinous process in open surgery, with one segment cranial to the cranial end of the surgical field. For percutaneous, minimally invasive surgical cases, a small midline incision cranial to the surgical field was performed, with a subperiosteal preparation of the spinous process, to allow for the fixation of the carbon reference array to the spinous process. 

In cases of the stabilization of multiple levels, the reference array was sometimes moved closer to the instrumented vertebra for better accuracy. For example, in cases for which stabilization Th10-L2 was performed, the carbon reference array was initially placed at Th9. Following the registration scan, robotic-assisted implantation of K-wires was performed in Th10, 11 and 12. Pedicle screws were then implanted under fluoroscopy control. The reference array was then moved to the spinous process of Th12, and a control CT scan was performed, covering the area of Th10-L2. This scan was used for implant-position control as alongside a registration scan for the stabilization of L1 and L2. 

A sterile coverage was applied on the patient, so that the reference array was still visible by the navigation camera. Following this, a navigated low-dose intraoperative CT scan, covering the region of interest (spinal segments which were planned for the stabilization), was performed. Immediately after scanning, imaging data was automatically transferred to the navigation system (BrainLab, Munich, Germany) without user interaction for automatic patient registration with the acquired image data [14]. 

**Figure 3:**
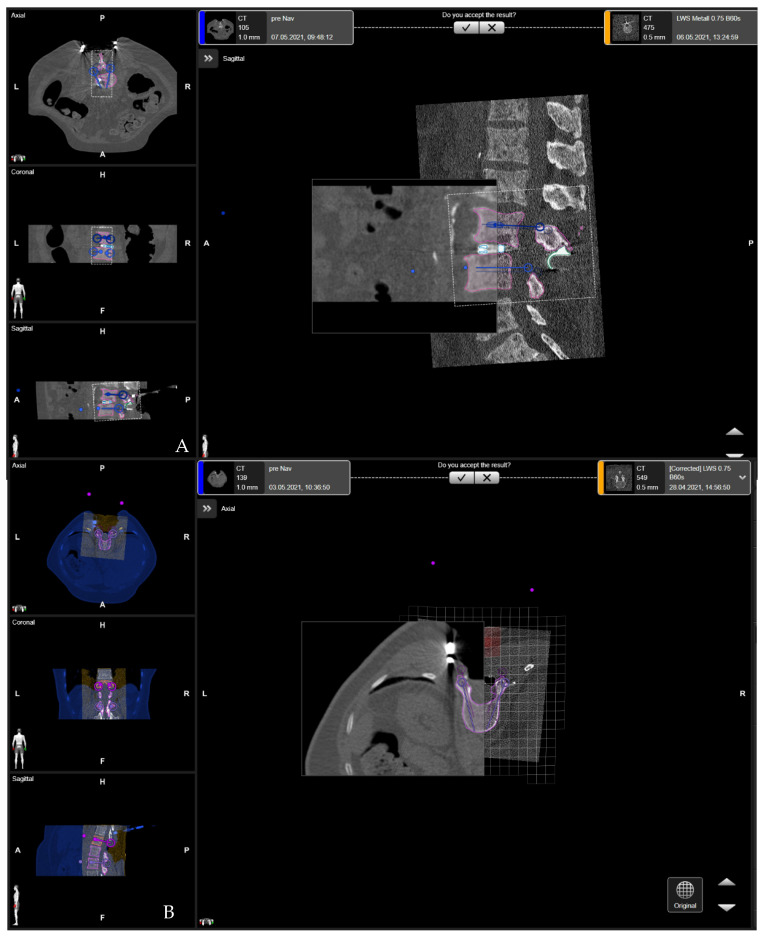
(**A**). Rigid fusion of the preoperative CT of the lumbar spine and registration iCT scan with preplanned screw trajectories (Patient number 3). (**B**). Elastic fusion of the preoperative CT of the lumbar spine and control iCT in the case of multisegmented stabilization in the thoracolumbar spine (Patient number 2).

To display preplanned screw trajectories in the recent imaging data, the co-registration of preoperative data, defining a region of interest with pre-planned screws, was conducted. Elements Spine Curvature Correction co-registers scans of the patient to compensate for inevitably varied spine positions during different imaging sessions. The software brings together scans from preoperative CT with iCT to update them for surgery. Additionally, if needed, preoperative magnetic resonance imaging (MRI) of the spine can be fused with preoperative and intraoperative imaging. Preoperative image data from CT and MRI are fused non-linearly, by applying the spine curvature element in a rigid and elastic fashion (rigid Elements Image Fusion 3.0 or elastic Elements Curvature Correction, Brainlab, Munich, Germany) [15].(Figure 3). Rigid fusion was only used in the region of segments which were planned for mono- and bi-segmental stabilization. Elastic fusion included the registration of the pre-aligned data and was used alongside rigid fusion in cases of multisegmented stabilization and for thoracolumbar constructs. 

**Figure 4:**
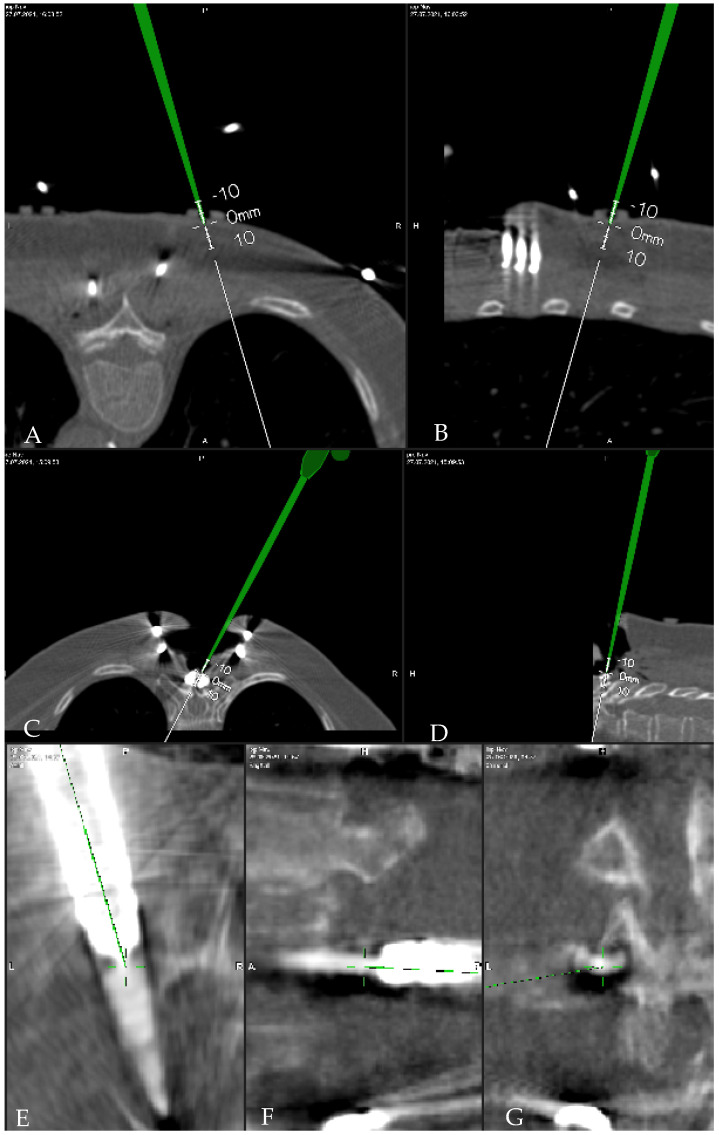
Registration accuracy check with tip of the pointer on (**A**,**B**) the skin fiducials (**C**). holder of the carbon reference array (**D**). the mini screw attached to the spinous process. (**E**–**G**). head of the screw.

Registration accuracy was evaluated by placing the pointer tip on anatomical landmarks such as the spinous process, vertebra lamina, or the vertebral body surface, or on artificial landmarks, such as skin fiducials or mini-screws attached to the spinous process [15]. A registration accuracy check can also be performed following control iCT with the pointer tip placed on the implants (Figure 4). For calculation of the effective dose (ED), the total dose length product (DLP) was multiplied by ED/DLP conversion factors, which are estimated to be 17.8 μSv/Gy × cm for thoracic, and 19.8 μSv/Gy × cm for lumbar scans [16,17]. The DLP refers to a phantom with a diameter of 32 cm for thoracic and lumbar examinations. 

An intraoperative CT was used for the robot-guided implantation of K-wires into the pedicles following the insertion of pedicle screws under fluoroscopy control. 

**Figure 5:**
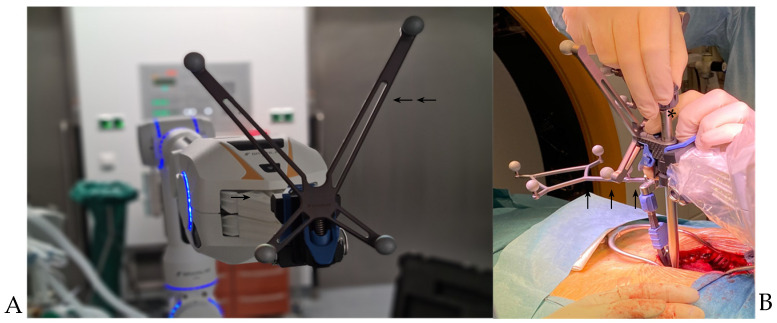
CIRQ Robotic Alignment Module. (**A**). Kinematic unit (arrow), tracking array (double arrow). (**B**). Intraoperative view. Attachment of drill guide (star) onto the kinematic unit. Reference array attached to the spinous process (triple arrow).

**Figure 6:**
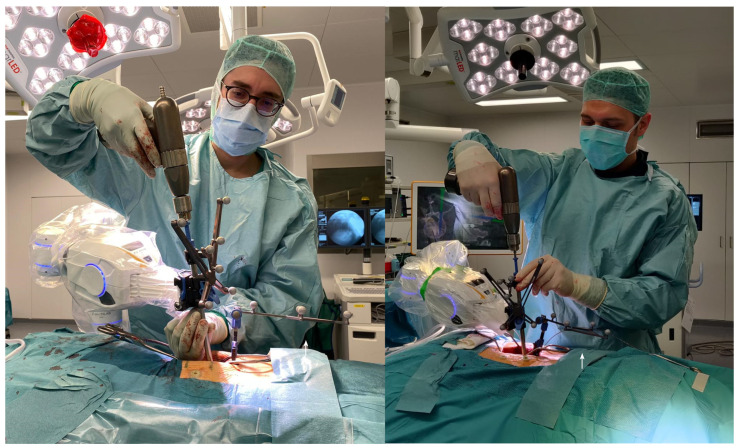
Position of the surgeon during the procedure. Reference array (arrow).

The Cirq^®^ Robotic Alignment Module consists of an interchangeable head, which enables alignment with the preplanned trajectory and a kinematic unit for fine-tuning adjustment, based on pre-planned trajectories, during computer-assisted robotic surgery. A tracking array was attached to the kinematic unit for real-time tracking of the instruments and constant position feedback (Figure 5). The first step is to move the robotic arm over the entry point of the preplanned trajectory. When the robotic arm has been roughly placed over the entry point of the trajectory, the robotic alignment module enables the automatic positioning of the robotic arm according to preplanned screw trajectory. A drill guide is then inserted through the tracking array and positioned onto the entry point; following this, the tracking array can be safely locked, which further secures the drill guide. Snap-on depth control is attached to the proximal part of the drill guide for precise and safe drilling. Following drilling, a K-wire is implanted, and the robotic arm detached. (Supplemental Material: Video). Here, the surgeon stood on the left side of the patient, next to the robotic arm (Figure 6).

A CT scan was performed at the same time as the scan for the control of implanted pedicle screws and a registration scan for subsequent stabilization. Following the implantation of K-wires, pedicle screw implantation was performed under fluoroscopy control. After screw placement, an intraoperative CT scan was performed to confirm correct screw placement.

The following parameters for the assessment of the initial experience were used for evaluation:

(a) Surgery time was defined as the period between the first incision and closure.
(b) The time taken for positioning and robot installation was defined as the time necessary to position the patient and completely install the robotic arm.
(c) The robotic time, which was recorded by the robotic system, was the total time in which the robotic arm was in use. This period covers the point of initiation of the first entry point search to the implantation of all K-wires.
(d) Time per screw—since we did not measure time needed for the implantation of each screw separately, time per screw was calculated per case by dividing the robotic time with the number of implanted screws. This provides a measurement of the time needed for robotic-assisted implantation of K-wires. Since K-wire implantation is the essential robotic-supported element of pedicle screw implantation, we labeled this time as time per screw.
(e) Pedicle size—measurement of the pedicle size in the axial, coronal and sagittal plane, on the registration iCT scan, with a line perpendicular to the preplanned screw
(f) Pedicle screw accuracy—measured according to the CT-based Gertzbein and Robbins System (GRS). The grading system reflects the deviation of the screw from the “ideal” intrapedicular trajectory. The transpedicular screw position was graded from A to E, based on the extent to which the screw breaches the cortex of the pedicle 1–3:
  - entire intrapedicular position without a breach of the pedicle cortex
  - exceeding the pedicle cortex < 2 mm
  - exceeding the pedicle cortex 2–4 mm
  - exceeding the pedicle cortex 4–6 mm
  - exceeding the pedicle cortex > 6 mm or reaches outside of the pedicle.

Grade A and B can be considered as satisfactory operation results. In grade C to E, neurological symptoms may occur and therefore, can be evaluated as an unsatisfactory surgical result [18].

(g) Deviation of preplanned trajectory from actual pedicle screw position—offset of the screw compared to preplanned trajectory (degrees to medial/lateral): mean deviation in entry point, the average deviation from the tip of the screw, and angular deviation. Deviations were determined using an image-overlay analysis to compare preoperative CT imaging with preplanned screw trajectories to a true screw position on intraoperative, control CT imaging (Figure 6). The mean deviation of entry point and the average offset from the tip of the screw were measured in the axial plane by determining the perpendicular distance of the midline of the planned screw versus the midline of the actual screw position; this latter line was drawn manually in the software as a best estimate on the slice with the widest screw diameter. Angular deviation was measured by determining the angle of the midline for the planned screw versus the midline of the actual screw position in the lateral plane.

**Figure 7:**
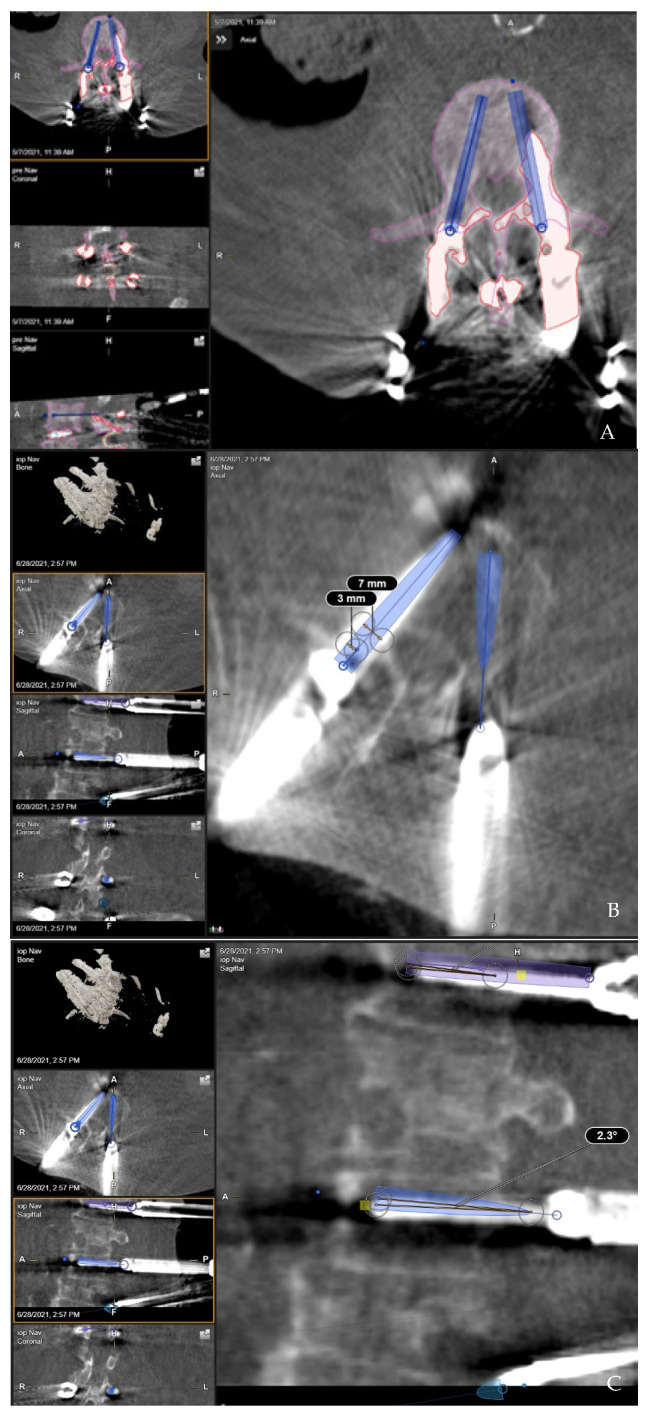
Accuracy of the implanted pedicle screws and deviation compared to the preoperatively planned screw trajectories were measured using the image overlay technique following rigid and elastic fusion of the preoperative CT scan, registration iCT scan and control iCT scan. (**A**). Implanted screws are segmented in red and the trajectory of the preplanned screws in blue. Entry point and tip point deviation were measured in the axial plane. (**B**). Overlay of a preplanned screw trajectory with the implanted pedicle screw. Entry point deviation 3 mm to medial and lateral from the entry point of the preplanned screw trajectory was considered acceptable for correct pedicle screw placement. (**C**). Angular deviation was measured in the lateral plane via the angular measurement tool in the BrainLab software using the measurement of the angle between the axis of the implanted screw and the preplanned screw trajectory.

Figure 7 is from the low dose iCT, these are the original resolution data.

### 2.2. Statistical Analysis

The analyses were performed using SPSS statistical software, version 20 (SPSS Inc. IBM, 1 Orchard Rd, Armonk, NY, USA). A *p* value of <0.05 was considered to be statistically significant. For variables such as deviation of the entry point, screw tip and angular deviation, the mean was calculated with standard deviation (SD). A *t* test was used to measure statistically significant difference between the means. For calculating the differences between standard deviations, Leven’s Test for Equality of variances was performed prior to the *t* test. In cases where a statistically significant difference between the SDs was found, a *t* test was not performed. For comparison, we decided to compare roughly the first half of all implanted screws with the second half. In the first 7 surgeries there were 36 and in the following 6 surgeries a total of 34 implanted screws. An independent sample *t* test was used for a comparison of different mean values between the first 7 and the other 6 surgeries, i.e., between the first 36 and the remaining 34 screws. 

## 3. Results

In total, 12 patients (5 male and 7 females, mean age 67.4 years) underwent 13 surgeries using the CIRQ Robotic Alignment Module for thoracolumbar pedicle screw implantation. One patient with spondylodiscitis underwent two surgeries for spinal fusion in the thoracic and lumbar spine (patient number one). Indications included spondylodiscitis (4 patients), metastases (3 patients), osteoporotic fracture (2 patients), and spinal canal stenosis (3 patients). A total of 70 screws were implanted. Six surgeries were performed using an open technique and the other 7 were performed using a minimally invasive percutaneous technique. The mean height of all patients was 171.4 ± 13.9 cm (range 145–189), with mean weight of 84.6 ± 23.2 kg (range 59–130). All patients underwent a registration CT scan for automatic navigation registration as well as at least one control CT scan for implant position control. The general characteristics of the patients are summarized in Table 1.

### 3.1. Operative Setting of the Patients

Patient 1. was admitted with severe spondylodiscitis of the cervical, thoracic, and lumbar spine. He initially underwent a stabilization on the cervicothoracic spine and decompression with empyema evacuation. Following this surgery and initial recovery, seethe was determined for stabilization in the thoracic and lumbar spine. The first surgery was performed using the open technique; the reference array was attached at the spinous process of Th8 for Th9/10 stabilization. Following a registration iCT scan, four K-wires were implanted along the preplanned screw trajectories in Th9 and Th10, followed by implantation of the pedicle screws under fluoroscopy control. Control iCT was performed and indicated the correct position of the implanted screws. In the second surgery, also using an open technique, the reference array was attached to the Th12 spinous process for LW1/2 stabilization; this was followed by XLIF-Surgery L1/2 in the right lateral decubitus position. 

Patient 2. underwent surgery Th11-L2 for spondylodiscitis. An open technique was used, and the reference array was attached at the spinous process of Th11. Following registration iCT, four screws were implanted using CIRQ Robotic Alignment, after control iCT, which confirmed the correct screw position. 

Patient 3. presented with spondylodiscitis L3/4 following dorsal surgery with interspinous spacer implantation. In the open technique, after registration iCT, robotic-guided screw implantation in L3 and L4 was performed, followed by control iCT with the correct placement of the screws. A registration array was attached at the L2 spinous process. The registration accuracy could have been monitored well using the interspinous spacer. Following this, a laminectomy and removal of the spacer was performed.

**Figure 8:**
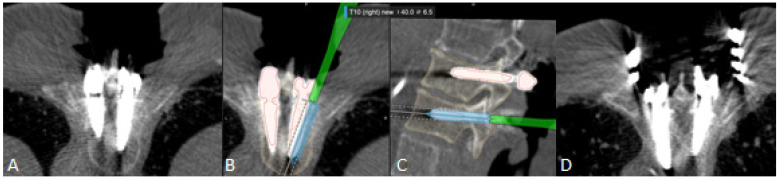
Intraoperative Th10 screw revision in patient number 4. After registration iCT, robotic-guided stabilization was performed for Th9-10 and control iCT scan was performed. (**A**). Axial view of control iCT shows GRS grade D Th10 screw on the right side with correct position of all other screws. The screw was removed and then repositioned using robotic alignment. (**B**). axial and (**C**). sagittal view of the screw revision. Screws were segmented for better visualization and orientation. The screw was implanted along the presented trajectory (blue). (**D**). Axial view of control iCT shows GRS grade A Th10 screw on the right side.

Patient 4. underwent stabilization for breast-cancer metastases through a compression of the spinal cord in Th 12. An open technique was used because a laminectomy and a partial resection of the tumor in Th12 were performed following screw implantation. Since a longer construct was planned, the reference array was initially attached to Th8. After registration iCT, robotic-guided stabilization was performed for Th9-10, and a control iCT scan was performed. This scan demonstrated a GRS grade of D for the screw in Th10 on the right side, with the correct placement of all other screws. A registration accuracy check confirmed the high accuracy of the scan. The proposed mechanism leading to the misplacement of the screw is a medial breach of the corticalis of the pedicle, due to tumorous damage of the bony substance (skiving) since the entry point was correct. The screw was repositioned using robotic alignment. A reference array was then attached at Th12 to use the following scan at the same time as control iCT for the repositioned screw and as registration iCT for the lumbar screws. In the iCT scan, the correct position of the repositioned screw was observed (GRS A screw). Robotic-guided stabilization L1/2 was performed; a control iCT scan revealed a grade D screw in L2 on the left side, with the correct placement of all other screws (grade A). An accuracy check demonstrated suboptimal accuracy, possibly due to the movement of the reference array attached to the spinous process of Th12, which was infiltrated by the tumor and therefore unstable. We experienced a further technical issue at this point with the CIRQ Robotic Alignment, so the L2 screw on the left side was repositioned using iCT-based navigation, following another iCT scan with the reference array repositioned on the L1 spinous process. Following screw revision, the newly placed screw was of a GRS grade of A (Figure 8.)

Patient 5. underwent stabilization using the MIS technique for breast cancer metastasis of the L1 with Th12-L2; a reference array was attached to Th12 spinous process via small separate midline incision. Following robotic-guided screw implantation, a transpedicular biopsy of L1 from the left side using iCT-based navigation was performed.

Patient 6. underwent MIS stabilization for multiple metastases on the thoracolumbar spine with instability. The reference array was attached at Th10 with a small separate incision; registration iCT and robotic-guided stabilization of Th11-Th12 was performed. The registration array was re-attached to L1, and iCT confirmed the correct placement of all screws, which was then used for the automatic registration of L2 and L3 screws. Following implantation of the screws in L1/2, a further iCT scan was performed, which showed GRS grade A screws in L1 left and right and L2 right with GRS Grade C L2 screw on the left side, with a lateral breach of less than 4 mm. This screw was not repositioned. 

Patient 7. underwent Th11-L2 stabilization through the MIS technique using robotic guidance; all screws were placed correctly. The patient also underwent corpectomy of L2 due to massive destruction caused by infection and implantation of the expandable cage, using the lateral approach.

Patient 8. underwent a stabilization using the open technique for spinal canal stenosis with instability. The reference array was attached at L2; control iCT confirmed the correct position of all screws. Further decompression with TLIF-Cage implantation was performed in L3/4 and L4/5.

Patient 9. presented with an L2 fracture including further severely osteoporotic changes in L4, leading to MIS-pedicle screw implantation in L1-3-5. The reference array was attached via separate incision on Th12. Following robotic-guided screw implantation, two control iCT scans were performed, since the first scan did not encompass the L5 level. All screws were placed correctly.

Patient 10. underwent stabilization in MIS technique L5/S1, following spinal canal stenosis and instability after two surgeries for a herniated disc at L5/S1 on the left side. The reference array was attached via a separate small midline incision on L4. After control iCT confirmed the correct position of all screws, the reference array was removed, and the incision was extended so that the subperiostal preparation of L5/S1 level on both sides was performed. Decompression of L5/S1 with tranforaminal interbody lumbar fusion (TLIF)-Cage Implantation was subsequently performed.

Patient 11. underwent thoracolumbar stabilization of Th10-L2 due to a previous Th12 fracture, and experienced a further fracture of L4 with adjacent segment disease in L2/3. In open technique, the reference array was attached at L1 and registration iCT was performed. Using robotic guidance, L3, L5 and S1 levels received screws, which all showed correct position. A decompression of L3-S1 was performed due to spinal canal stenosis. Loosened L2 screws were removed, and the newly implanted screws connected the Th10-L1 screws with one rod.

Patient 12. experienced a Th12 burst fracture with maximal spinal canal stenosis. Stabilization using the MIS technique was performed for Th10/11 on L1/2; the reference array was attached via a separate incision on the spinous process of Th12. A control iCT scan confirmed a GBS Grade of A for the position of all screws. In a secondary surgery, corpectomy of Th12 was performed via the lateral approach, with implantation of the expandable cage. 

Patients number 9. and 11. had paraparesis which did not improve following surgery. Patient number 12. had a severe ataxia which slightly improved following surgery. All other patients did not have neurological deficits prior to surgery and underwent thoracolumbar stabilization due to intractable pain and instability in the spine due to infection or tumor. 

Complications occurred intraoperatively in patient number 4., where two screws were intraoperatively revised. The patient did not experience any neurological deficits following surgery. Patients number 6. and 8 experienced wound-healing deficits due to seroma, which required additional surgery. Patient number 7. developed wound healing problems on the lateral, XLIF wound, and needed a revision surgery. Patient number 11. developed a CSF leak, which required revision surgery. 

The mean surgery time was 03:37:14 ± 01:30:29 h.

The mean positioning and robot installation time was 53:37 ± 36:46 min. The mean time for the first 7 surgeries was 64:43 ± 45:9 min and for the latter 6 surgeries the mean time was 40:6 ± 12:9 min. This difference was not significant (*p* < 0.05).

The mean robotic time was 45:40 ± 34:29 min. The mean robotic time for the first 7 surgeries (36 screws) was 76:18 ± 35:10 min and for the latter 6 surgeries (34 screws) 25:14 ± 10:45 min; this difference was considered to be significant (*t* test, *p* < 0.05). The mean robotic time for open surgery was 52:01 ± 41:30 min and for MIS surgery 42:56 ± 30:36 min; this difference was not significant.

The mean time per screw was 08:27 ± 06:54 min. The mean time per screw for the first 7 surgeries (first 36 screws) was 16:03 ± 09:32 min. The mean time per screw for the latter 6 surgeries (34 screws) was 04:35 ± 02:11 min. This difference was significant (independent *t* test, *p* < 0.05).

All patients were operated on by a team of two surgeons. Patients number 1,2,7,8 and 11 were operated on by a senior surgeon (senior author B.S., with 5 years of experience in complex spine surgery) and patients number 3,4,5,6, 10 and 12 by surgeon M.P. (corresponding author, with 3 years of experience in complex spine surgery). Patient number 9. underwent surgery by both surgeons. The mean surgery time for surgeon B.S. was 03:06:50 ± 01:22:38 h, and the mean surgery time for surgeon M.P. was 04:20:10 ± 01:21:34 h. Excluding patient 9., the mean time per screw for surgeon B.S. was 11:35 ± 09:57 min and the mean time per screw for surgeon M.P. was 10:56 ± 08:44 min. Patient number 4, who underwent revision for two screws, was operated on by a surgeon with less experience (M.P.).

Patients underwent one registration iCT and one control iCT scan. Patient number 9. underwent 3 iCT scans and patient number 4. underwent 4 iCT scans.

### 3.2. Pedicle Screw Accuracy

A total of 70 pedicle screws were implanted using Cirq^®^ Robotic Alignment, with an average of 5.38 screws placed for the patients and a mean of 2.46 ± 1.21 levels fused. A total of 44 screws were implanted in the lumbar and 22 screws in the thoracic spine; there were four screws implanted in the sacrum (S1). The most common screw dimensions were 6.5 × 50 mm (26 screws) and 6.5 × 45 mm (24 screws), followed by 6.5 × 40 mm (8 screws), 6.5 × 60 mm (4 screws), 5.5 × 50 mm (4 screws), 6.5 × 55 mm (2 screws) and 7.5 × 40 mm (2 screws). There were no perioperative neurological deficits.

The mean axial pedicle size measured on the registration scan was 11 ± 4 mm, mean coronal 12.1 ± 2.9 and mean sagittal 12.1 ± 2.9. In the thoracic spine, mean axial, coronal and sagittal size were 8.5 ± 1.9, 11.9 ± 1.37) and 10.2 ± 1.33 mm, respectively. In the lumbosacral spine, mean axial, coronal and sagittal size were 12.25 ± 4, 12.3 ± 2.2 and 13 ± 3 mm, respectively. 

Mean entry point deviation ranged from 0.5 to 5 mm and was 1.9 ± 1.23 mm for all screws (2 ± 1 for thoracic and 1.84 ± 1.31 for lumbosacral spine). 

The mean deviation from the tip of the screw ranged from 0 to 7 mm and was 2.61 ± 1.6 mm for all screws (3 ± 1.5 mm for thoracic and 2.41 ± 1.6 mm for lumbosacral spine). 

Angular deviation ranged from 1° to 9.30° and the mean was 3.5° ± 2° for all screws (3.4 ± 2° for thoracic and 3.5 ± 2° for lumbosacral spine). 

For screw placement accuracy, we used the CT-based Gertzbein and Robbins System (GRS). Out of 70 screws, 68 screws were placed in a way that intraoperatively revision was not required. Of these screws, 65 screws were GRS A screws (92.85%), one screw was GRS B screw with a lateral breach of less than 2 mm (1.4%) and the remaining two screws were of C grade, with a lateral breach of less than 4 mm (2.85%). Two screws were D screws (2.85%), with a medial breach of more than 6 mm. These screws underwent intraoperative revision. One screw was revised using the robotic arm and one further screw was revised using iCT-based navigation with robot abandonment. This was also the only case of abandonment of the robot (Patient Number 4.). Following revision, both mispositioned screws were found to have a GRS grade of A. 

For the 70 screws placed, a rate of non-revised screws of 97.1% was recorded (GRS grade A/B/C screws), with 68 correctly positioned screws without need for revision. There were two mispositioned screws in one patient, which required revision surgery, both with a medial breach, one of which was repositioned using the robotic arm, and the other was repositioned using iCT-based navigation. 

## 4. Discussion

### 4.1. Previous Studies on Use of CIRQ^®^

So far, only a few studies examining the novel robotic system Cirq^®^ Brain Lab Munich, Germany have been published. The first known study provided a 2D-surgical video, depicting the surgical workflow with the O-arm and instrument holder module of the robot [19]. There are two forms of modular robotic assistance for drill stabilization, namely, an Instrument Holder Module and Robotic Alignment Module. While the instrument holder module provides stable support for drill stabilization, it requires an operating surgeon to place the robotic arm at the precise position of the preplanned screw trajectory. A Robotic Alignment Module, on the other hand, requires that the surgeon places a robotic arm over the entry point of the preplanned screw trajectory; following this, the robotic arm automatically aligns to the preplanned trajectory and, in this manner, minimizes the potential error. 

Two further studies include the application of the Cirq^®^ Instrument Holder, the first in the cervical spine to achieve minimally invasive C1-C2 stabilization for an odontoid fracture [20], and the second in a case series on its application for cervical and upper thoracic pedicle screws from the same working group [21]. In their case series, Farah et al. reported a high rate of poorly placed screws of 14% [21] although with excellent postoperative course. The radiation dose received by the patient was 9.1 mSv (range, 7.7–10.6 mSv) with 0 mSV exposure for the staff [21]. The literature is comparatively sparse on the use of robotic assistance for implants in the cervical spine, likely due to concerns surrounding the relatively significant intraoperative mobility of the cervical spine, which risks impacting the accuracy of a robot’s navigation system [20]. The lightweight and table-mounted aspects of the new Cirq^®^ arm are intended to be more ergonomic and less disruptive to operative workflow relative to larger robotic units [20]. Furthermore, reports show Cirq^®^’s potential cross-compatibility with implants from multiple manufacturers [20].

### 4.2. Previous Studies on Use of Other System for Robotic-Guided Spine Surgery

MAZOR Spine Assist was the first robotic system to be approved for use in spine surgeries in the USA in 2004 [22]. Prior work examining the accuracy of screw placement using robotics is promising, with rates ranging from 85% to 100% [23]. Navigation-enabling technology such as a 3D-platform (O-arm) or intraoperative mobile CT (iCT-Airo) systems for use in spinal surgery has considerably improved accuracy compared to traditional fluoroscopy-guided techniques during pedicular screw positioning [24]. The introduction of a mobile CT scanner reduced the rate of screw repositioning, which enhanced patient safety and diminished radiation exposure for patients, but it did not improve overall accuracy compared to that of a mobile 3D platform [24].

Most spinal surgery robots are shared-control robots that simultaneously provide both the surgeon and robot the ability to control instruments and motions [25]. There are several studies evaluating the utility of pedicle-screw implantation using robotic-guided spine surgery with different systems, such as, Mazor X, Mazor Robotics Ltd., Caesarea, Israel [26], Mazor [27], SpineAssist Mazor [9,12,28,29,30], Excelsius GPS^®^; Globus Medical, Inc. Audubon, PA, USA [2,31,32], ROSA spine robot technology (Zimmer-Biomet, Warsaw, Indiana, USA) [33], Orthbot [34] and TiRobot [35]. Unfortunately, direct comparisons of screw placement accuracy between these systems are difficult because of the significant cost and time associated with their adoption [36]. Most studies in the literature evaluating the accuracy of robotic-assisted screw placement utilized one of the Mazor platforms, with reported accuracy in the range of 97–99% using the Gertzbein–Robbins grading system [37,38,39]. The accuracy of traditional pedicle screw placement appears to be in the same range of 97–98% [2,40].The ROSA robot system has been evaluated in feasibility and cadaveric studies, as well as in a small number of patients, making its comparison to other robotic technologies difficult [6,41]. In our initial experiment with CIRQ^®^, used on a small number of patients, 97.1% of screws did not require intraoperative revision. This rate is slightly lower than the reported accuracy of existing robotic systems. However, this initial study provides guidance on operative workflow and aimed to identify and prevent possible events that could lead to less accuracy in pedicle screw placement.

Cahil et al. described the operative workflow of Mazor Spine Assist [42]. Most commonly, the surgeon attaches the platform to the patient’s spinous processes using one K-wire, before using two additional K-wires to secure the platform to the patient bilaterally, while for minimally invasive procedures, the robot is attached to a frame supported by a percutaneously placed guide wire [43]. Studies conducted by Hu et al. [38] and Ringel et al. [44] used this robotic system, the latter using a percutaneous approach with one K-wire attached to the spinous process and two Steinmann pins attached to the posterior superior iliac spines [44]. In both studies, the authors observed instability in the K-wire, leading to mispositioned drill sleeves and skidding of the drill cannula as well as difficulties involving tool skiving and trajectory completion. A randomized controlled trial using ROSA SpineAssist robot found that 93% of the pedicle screws placed with the freehand technique were Gertzbein-Robbins A or B compared with 85% for those placed with robotic guidance [44]. In contrast, a meta-analysis that included 10 studies with different robotic systems found robotically assisted pedicle screw placement performed better than freehand screw placement in terms of “perfect accuracy” and were characterized as more “clinically acceptable” [45]. Using SpineAssist™, a spinous process-mounted miniature robot, Fan et al. significantly reduced adverse events, fluoroscopy time per screw, postoperative stay, and blood loss compared to navigation template or O-arm systems, in their analysis of 890 pedicle screws placed in 190 adult patients using four different techniques [46].

The Renaissance^®^ is the Mazor’s second-generation spine robot, replacing the SpineAssist in 2011, with upgraded image recognition algorithms and the ability for the surgeon to flatten the bone around screw entry points before drilling for prevention of skidding for the guiding cannula on a sloped anatomy [11]. These well-known problems of the robotic-assisted spine surgery were also encountered in our case series. One possible mechanism is the movement of the reference array and therefore, the deranged navigation accuracy. Both SpineAssist and Renaissance have been found to result in accuracy rates ranging from 85% to 100% [47,48]. Mazor X^®^ is the most recent release by Mazor, and includes an integrated linear optic camera that allows the robot to perform a volumetric assessment of the work environment in order to self-detect its location and provide collision avoidance intraoperatively, allowing each vertebral body to be registered independently and hence has its own accuracy [11,49]. The automatic recognition of vertebra with automated screw planning is a feature enabled by CIRQ^®^. One prospective study which compared Mazor-X assisted pedicle-screw implantation to computerized-navigation based implantation of the pedicle screw has demonstrated that robotic technology exposed patients to a reduced fluoroscopy time, decreased time-per-screw placement and shorter hospital stay than 3D-CT navigation [26]. 

The Excelsius GPS System features real-time intraoperative imaging, automatic compensation for patient movement, and direct screw insertion through a rigid external arm–obviating the need for K-wires or clamps, with feedback provided instantly via the robot’s monitor if the drill skives or the reference frame moves [50]. Farber et al. described their initial experience with Excelsius GPS System [36]. Fayed et al. reported 103 PPS in the first 20 consecutive patients with postoperative computed tomography imaging using ExcelsiusGPS Robot 6 breaches, with only two breaches >2 mm, yielding an overall breach rate of 5.8% and a significant breach rate of 1.9%. In comparison, their fluoroscopy-guided cohort had a breach rate of 3.3% and a significant breach rate of 1.1%, which was not significantly different [51] Of the 600 pedicle screws inserted by navigated robotic guidance (101 patients), only 1.5% (9/600) were repositioned intraoperatively in the study by Vardiman et al. using the same robotic system (Excelsius GPS); this study demonstrated a high level of accuracy (based on GRS) with no significant differences between the left- and right-side pedicle screw placements (98.67% vs. 97.67%, respectively) in the clinical use, whereas screws on the left were placed by resident and screws on the right by attending surgeon [52]. Overall, 2/70 screws required intraoperative revision in our case series. Further assessment, using a greater number of implanted screws, is needed, since the first cases, when using a novel robotic system, are prone to adverse events such as screw malposition due to registration inaccuracy, movement of the reference array or screw skiving.

ROSA^®^ SPINE was approved by the FDA in 2016. Similar to Mazor X, the free-standing ROSA utilizes a robotic arm and navigation camera—each mounted to their own floor-fixable mobile bases—in order to optimize and guide pedicle entry points and trajectories [53]. Lonjon et al. reported the screw placement accuracy using this robotic system to be 93.7%, compared to 92% in the freehand group (36 vs. 50 screws) [53]. Operative workflow and the setup of the newly appeared Mazor X Stealth Edition (MXSE), with robotic-assisted cortical bone trajectory (CBT), was recently published [37]. Currently, there are several novel robotic systems for which utility, operative workflow and pedicle screw accuracy need to be assessed, namely, NuVasive Pulse^®^ (San Diego, CA, USA), Curexo^®^ (Curvis-spine, South Korea) and Fusion Robotic System^®^. 

### 4.3. Surgery Time and Robotic Time/Pedicle Screw Insertion Time

The mean surgery time in our cohort was 03:37:14 ± 01:30:29 h and differed only slightly between open and MIS surgery. A long operative time is attributed to the decompression of the spinal canal stenosis, discectomy, cage implantation, tumor resection or biopsy, which are unrelated to screw implantation. This is consistent with previously published results on the initiation of a robotic system for which the operative time was reported to be 224 min (193 to 307 min) in a study of Bydon et al. (Spine Assist TM^®^) [54]. In their study, increased surgical experience led to a significantly reduced operative time, which was not the case in our cohort [54]. A significantly longer mean operative time was noted in the robotic cohort than the freehand cohort in previous studies. Lonjon et al. attributed this to both learning curve and set-uptime for robotic use (ROSA^®^ Spine) [53]. A prolonged operative time was noted from several investigators [31,55]. Kantelhardt et al. reported an operative time of 140 to 254 min per robot-assisted case (Spine Assist TM^®^) [29]. A recent meta-analysis also showed that the length of surgery was significantly higher for robotic-assisted surgery compared to the freehand (FH)-group [56].

The mean robotic time was 45:40 ± 34:29 min. For the first 7 surgeries (36 screws) it was 76:18 ± 35:10 min and for the latter 6 surgeries (34 screws) it was 25:14 ± 10:45 min, with significant differences. Although measured on a small sample size, this improvement shows a certain learning curve. Unfortunately, we did not measure the pedicle screw insertion time for each screw given the workflow of implantation of the screw, where first a set of K-wires was implanted followed by serial implantation of screws under fluoroscopy. This is the reason why time per screw was measured as a division of robotic time through the number of screws for each case. Using a similar method of screw insertion time measurement (including time needed for implantation of all screws in one surgery), screw placement times for the latter 6 surgeries (25:14 ± 10:45 min) were found to be similar to a study by Feng et al. [57] and the study of Benech et al. (Excelsisu GPS^®^) [2] (27.60  ±  8.58 vs. 25.7  ±  14.2). 

The mean time per screw was 08:27 ± 06:54 min, and for the first 7 surgeries (first 36 screws) it was 16:03 ± 09:32 min, and for the latter 6 surgeries (34 screws) was 04:35 ± 02:11 min. This difference was significant. In a study that compared robot-guided and 3D-CT navigation with O-Arm, the mean time-per-screw placement was found to be 3.7 min for the robotic group and 6.8 min for the 3D-CT navigation group with fluoroscopy time, time-per-screw placement and length of stay significantly lower in the robotic group [26]. Robotic guidance achieved the following pedicle screw times: 4 min (Hyun et al. [7]), 3 min (Pechlivanis et al., percutaneous cohort) [8] and 3.6 min for a midline approach and 5.7 min for a percutaneous approach (Urakov et al. [58]) Hu et al. report that time per screw decreases and the learning curve plateaus after 30 cases (Spine Assist TM^®^) [38]. In our small case series, we observed a trend of decrease of robotic time and time per screw. In our case series, surgery time was longer for surgeons with less experience, whereas there was no significant difference between the time for screw between the two surgeons. These findings could have been biased by the fact that the senior surgeon performed the first three cases, where the robotic system was initialized, and the operative workflow was still under construction.

The mean positioning and robot installation time was 53:37 ± 36:46 min. The mean time for the first 7 surgeries was 64:43 ± 45:9 min and for the latter 6 surgeries 40:6 ± 12:9 min, with one case of robot abandonment. Robot abort time has been reported in up to 9.7% of cases [31] with a 12.5% manual screw placement rate [38].

### 4.4. Deviation of the Screws from the Preplanned Trajectory

The mean entry point deviation ranged from 0.5 to 5 mm and was 1.9 mm (SD ± 1.23), the average offset from the tip of the screw ranged from 0 to 7 mm and was 2.61 mm (SD ± 1.6), and angular deviation ranged from 1° to 9.30° and the mean was 3.5° (SD ± 2°). The mean entry point deviation is consistent with the findings of van Dijk et al., who, in a study on 112 robot-guided spine surgery patients, reported a mean deviation of entry point of 2.0 ± 1.2 mm, compared to preoperative plan (Spine Assist TM^®^) [12]. The same authors reported a mean difference in angle of insertion of 2.2° ± 1.7° on the axial plane and 2.9° ± 2.4° on the sagittal plane, which is lower than in our cohort [12]. However, data in the literature largely differ. Inaccurate screws (Grade C and D, respectively) in one series displayed a tip and tail deviation greater than 1.5 mm [2] and 57.1% of clinically acceptable screws (Grades A and B) had an offset of greater than 2° compared to 80% of inaccurate screws [2]. Godzik et al. found a 2.6  ±  1.5 mm tip offset, 3.3  ±  2.0 mm tail offset, and 5.6  ±  4.3° angular offset [31] with an average 5.0 ± 2.4 mm 3-D and 2.6 ± 1.1 mm 2-D error (Excelsius GPS^®^). Furthermore, an average 2 ± 1 mm error at the midpoint of the screw, which corresponded to the location of the pedicle, was observed [31]. One of the drawbacks of our study is that only the 2-D accuracy was measured. The authors report no differences in the 3-D or 2-D accuracy between breached and non-breached screws, and the breached screws had a plan that included less than 2 mm of the medial cortical wall. A significant portion of the breaches occurred in the thoracic spine, likely due to the smaller size and anatomy of the thoracic pedicle [12,31].

For screw placement accuracy, we used the CT-based Gertzbein and Robbins System (GRS). Out of 70 screws, 68 screws were placed so that intraoperative revision was not needed. Of these, 65 screws were GRS A screws (92.85%), one was a GRS B screw with a lateral breach of less than 2 mm (1.4%) and two further screws were grade C, with a lateral breach of less than 4 mm (2.85%). Van Dijk et al. found no correlation between GRS grading and screw offset from planned trajectory, meaning that a screw that deviates from the planned trajectory does not constitute inaccurate screw placement [12]. Jiang et al. described, in 47 patients and 253 screws, mean screw-tip accuracies of 1.3 ± 1.3 mm, 1.2 ± 1.1 mm, and 2.6 ± 2.2 mm in the mediolateral, cephalocaudal, and screw long axes, respectively, for a net linear deviation of 3.6 ± 2.3 mm and net angular deviation of 3.6° ± 2.8°. 

According to the Gertzbein-Robbins grading system, Jiang et al. reported that, in their series, 184 screws (72%) were classified as grade A and 70 screws (28%) as grade B [59]. The high degree of deviations reported the literature regarding screw deviation and classification of the screw position requires a re-evaluation of the grading system, to classify clinically acceptable screws, non-revised screws, and screws that require intraoperative (or postoperative) revision. Cadaver studies have shown that the use of robotic guidance decreased the number of placements in the “danger zone” (category D) by 72.2%, thus reducing the likelihood of injury to neurologic structures, which is a major risk related to screw misplacement [60].

Intraoperative complications pertaining to robotic guidance usually occur due to skiving of the screw and low entry points [26]. A meta-analysis of screw revision surgery revealed intraoperative screw revisions occurred for 8.8% of screw insertions in both robotic and CT navigation technique [61]. Devito et al., in their analysis of more than 840 cases with robot-guided spine surgery, observed neurologic deficits in 4 cases, yet, following revisions, no permanent nerve damage was encountered, in contrast to the 0.6% to 5% of neurologic damage reported in the literature [9].

Prolonged surgery time, especially in the first cases following the initiation of the robotic system in the department, could lead to a higher infection rate compared to fluoroscopy guided or navigation-guided pedicle screw implantation. However, Menger et al. conducted a large, retrospective study of 557 patients and found robotic surgery to be cost-effective, resulting in reduced length of stay, fewer revision surgeries, lower infection rates, and shorter operative time [62]. They describe how improved screw accuracy in robotic surgery would allow for the conversion of more cases to minimally invasive surgery (MIS), which could result in reduced length of stay and fewer post-operative infections. An infection rate of 4.6% for open surgery and 0.0% infection rate for MIS has been demonstrated, suggesting that robotic technology could save $36,312 from reduced infections alone [11,62]. Kantelhardt et al. found a significantly lower postoperative infection rate of 2.7% in robotic procedures vs. 10.7% in fluoroscopy procedures [29]. The study by Han et al. found no difference in the surgical site infection rate between the robotic and non-robotic group [63].

### 4.5. Comparison of Robotic-Guided Spine Surgery with Fluoroscopy-Guided and Navigation-Guided Spine Surgery

There were several studies that compared robotic guided with fluoroscopy guided implantation of pedicle screws. In their retrospective review on 70 patients with metastatic spine disease and a total of 406 screws, Solomiichuk et al. described a misplacement rate of 15.6% in the robotic and 16.4% in the fluoroscopy group, with no difference in surgical site infection, duration of surgery and radiation time [64]. Interestingly, Molliqaj et al., in their comparison of 439 robot-assisted vs. 441 conventional screws, reported that the proportion of non-misplaced screws (corresponding to Gertzbein-Robbins Grades A and B) was higher in the robot-assisted group (93.4%) than the freehand fluoroscopy group (88.9%) [65]. A recent systematic review found that there is insufficient evidence to unequivocally recommend one surgical technique over the another [66], since the duration of surgery did not differ, there are mixed data on radiation exposure, and the studies which favored robotic guided pedicle screw implantation in terms of placement accuracy often did not reach statistical significance. A prospective randomized controlled trial by Hyun et al. that compared the impact of robotic guidance in minimally invasive spine surgery (MIS) to a fluoroscopy-guided, open approach, in lumbar fusions, revealed that patient outcomes were not affected by the surgical technique, although MIS robotic guidance showed reduced radiation exposure and length of stay [7]. Marcus et al. identified several potential sources of bias when comparing robotic-guided and fluoroscopy-guided spine surgery: the selection bias (following the introduction of the robot into surgical practice, straightforward cases were selected in the first instance while the operating team was familiarizing themselves with the technique), for which there are potential confounders such as unbalanced age, sex and BMI, which are usually not taken into consideration, Most of the studies contained a mixed cohort of patients who underwent stabilization in the thoracic and lumbar spine and patients who underwent open and MIS surgery, whereby screw placement was not universally assessed and several grading systems were used [66]. A recent meta-analysis on 39,387 patients who underwent posterior lumbar fusion revealed that robotic-assisted fusion had similar rates of surgical and medical complications compared with those who underwent conventional fusion [67]. In contrast to this finding, Liounakos et al., in their single center evaluation of 374 robotic-guided and 111 fluoroscopy guided procedures, reported that robotic guidance was associated with significant reductions in postoperative complication rates at all follow-up time points and significant reductions in revision rates at 90 days and 1 year [68].

It has been argued that the accuracy of screw placement with the use of navigation is high enough, so that use of a robotic arm is not necessary. However, the possibility of screw planning prior to surgery can significantly decrease surgical time, maximize the fidelity of screw placement and mitigate the human error that is ever present in repetitive manual tasks [36]. A recent meta-analysis that included a randomized controlled trial and 5 comparative cohort studies consisting of 529 patients and 4081 pedicle screws, demonstrated that robotic-guided surgery (Mazor) has a significantly higher accuracy than computer-assisted navigation in terms of optimal and clinically acceptable pedicle screw insertions. Furthermore, the robotic technique showed significantly less blood loss but equivalent intraoperative times, complications and revision surgery caused by malposition [69]. Many studies showed that RG, with intrapedicular accuracy rates ranging from 78.8% to 91.3%, was remarkably more accurate than CAN, with accuracy rates that ranged from 66.0% to 84.1% [27,45,46,70]. However, other researchers [26,28] did not find a marked difference between the two techniques. By contrast, Perdomo-Pantoja et al. found that the CAN method had a superior insertion accuracy rate of 95.5% over the Mazor RG and freehand techniques [71].

As of yet, only one single-surgeon study has been conducted, which evaluated differences between O-arm navigation and Mazor robotic-guide navigation (46 vs. 39 cases), revealing that screw placement was significantly more accurate and precise with robotic assistance when considering Gertzbein-Robbins A placement whereas mean operative times, estimated blood loss, wound revision rates and clinically acceptable instrumentation placement did not demonstrate significant differences between the 2 groups [70]. Laudato et al. did not find any differences in screw accuracy between the groups of freehand, navigated and robotic-guided surgery in their analysis of 569 screws; however, in this study, only 64 screws were implanted with robotic assistance [27].

### 4.6. Disadvantages of Robotic-Guided Spine Surgery

No clear economic benefit of robotic systems has been shown. Key elements that impact economic feasibility include the efficient use of surgical time and a decrease in revision and complication rates [31]. The high initial time burden associated with a surgeon’s use of robotic technology will continue to have implications in operating-room utilization time and cost-effectiveness [31]. The learning curve and initially longer operating times are well recognised disadvantages of robotic surgery [72]. Lonjon et al. found a significantly longer mean operative time in the robotic cohort than the freehand cohort, and attributed this increased time to both the learning curve and set-uptime for robotic use [53]. A significant improvement in estimated intraoperative blood loss was observed with a decrease in time in anesthesia, surgery, and robotic usage as well as a decrease in pedicle screw-insertion time and operative time [13]. The complication rate did not show any learning curve [13]. Technical issues such as registration and installation issues, a software bug and a placement issue have been described [13]. As previously mentioned, robot abort time has been reported in up to 9.7% of cases [31] with a manual screw placement rate of 12.5% [38]. Potential areas that could influence operative time as well as the installation process include changes in the team, including staff, residents and scrub nurses [13].

### 4.7. The Learning Curve

The learning curve is largely dependent on experience and the dedication of the operator [58]. For complications and misplacement of screws, no learning curve was identified [13]. Khan et al. describe only a minimal learning curve [49]. Van Dijk et al. did not find any learning curve in screw accuracy and screw deviation [12]. Siddiqui et al. described their initial experience using ExcelsiusGPS Robot from Globus Medical in 120 patients, and found that both the experienced surgeon and first fellow displayed a learning curve and achieved a statistically significant improvement of accuracy after 30 screws and that the second fellow had significantly better accuracy than the experienced surgeon in his first 30 screws [73]. Urakov et al. did not find any statistically significant differences when analyzing robot operators’ years of experience (Renaissance^®^) [58]. In addition to the surgeons’ learning curve, the team itself (including scrub nurses, company representatives, residents, and surgeons) need to be trained more effectively in order to decrease the “failure” rate [13]. This includes the scrub nurses and residents, especially for installation processes, such as the mounting of the device over the operating room table [13]. The learning curve for pedicle screw placement in terms of time and complication rate is minimal for an experienced surgeon with significant improvement of anesthesia time, surgery time, and blood loss [13]. This suggests that the major improvement in time is related to the learning curve of the operating team, including, but not limited to, surgeons, nurses, those tasked with surgical positioning, and company representatives for optimizing the timing of installation of the robotic system [13].

### 4.8. Limitations

There are several limitations of our study. First, this is a retrospective review of prospectively archived data. Patients comprise a heterogenous group for various indications using open surgery and the minimally invasive technique. We did not have a control group but compared our data to the data from the literature. A comparison of the initial experience of our study, using robotic-guided spine surgery with several hundreds of cases, a free-hand/fluoroscopy guided technique and CT-based navigation, performed by the two surgeons, did not seem practical. We selected relatively easy cases for the robotic-guided implantation of pedicle screws to first gain experience with a novel robotic system, with the intention to use the robotic arm for more complex cases in the future. In future studies, we plan to use the robotic arm for complex cases, including scoliosis surgery and revision. Furthermore, after our initial experience and set up of the operative workflow, further studies that compare pedicle screw implantation in more complex cases using iCT-based navigation vs. robotic-guided pedicle screw implantation are needed, to validate the value of the new operative method compared to the surgical standard. We did not measure the pedicle screw time but the total time for which the robotic arm was in use, divided by the number of implanted screws. Furthermore, an assessment of the accuracy of the pedicle screw implantation was performed in only a 2-dimensional fashion. However, this initial experience described the workflow and gave rise to relevant observations on the operative setting. Prospective studies that compare CT-navigation-based pedicle screw placement and the placement of pedicle screws using a robotic arm are needed for the evaluation of the efficacy of the Cirq^®^ Robotic Module. This data provides valuable initial benchmarks for comparison and lessons for wider scale implementation [31].

## 5. Conclusions

The Brainlab’s Cirq^®^ Robotic Alignment surgeon-controlled robotic arm is a safe and beneficial method for accurate thoracolumbar pedicle screw placement with high accuracy. The high accuracy observed in this study is consistent with the data from the literature. A learning curve was observed through the reduction of the robotic time and time per screw compared to total surgery time. Prospective studies are needed for a better evaluation of the novel robotic system. An optimization of the workflow should be considered for a reduction in the surgery time. 

## Figures and Tables

**Table 1:** General characteristics of the patients.

Patient	Age	Gender	Diagnosis	Open/MIS Pedicle Screw Implantation	Levels of Fusion	Non-Screw Surgery	Positioning and Robot Installation Time (Minutes)	Surgery Time (Minutes)	Robotic Time (Minutes:Seconds)	Time per Screw (Minutes:Seconds)
1.	70	Male	Spondylodiscitis	First surgery: Open Second surgery: Open	First surgery: Th9-10 Second surgery: L1-2	First surgery: None Second surgery: L1/2 stabilization via dorsal approach and XLIF 1/2	First surgery:107.23 Second surgery (dorsal approach): 62	First surgery:200 Second surgery: 116	First surgery:118:20 Second surgery: 80	First surgery: 29:35 Second surgery: 20:00
2.	50	Female	Spondylodiscitis	Open	Th11-L2	None	12	135	15:10	3:48
3.	74	Male	Spondylodiscitis	Open	L3-4	Laminectomy, Removal of spacer L3/4	60	360	108:40	27:10
4.	76	Female	Breast cancer metastases	MIS	Th9/10-L1/2	Laminectomy Th12/L1	55	352	111:10	13:53
5.	61	Female	Breast cancer metastases	MIS	Th12-L2	Transpedicular biopsy L1	54	142	57:40	14:25
6.	59	Female	Breast cancer metastases	MIS	Th11/12-L2/3	Transpedicular biopsy L1	40	240	28:10	3:31
7.	79	Male	Spondylodiscitis	MIS	Th11-L2	Corpectomy and implantation of expandable cage L1	45	180	33:07	8:16
8.	60	Male	Spinal canal stenosis	Open	L3-5	Decompression and TLIF Cage L3/4 and L4/5	45	360	37:02	6:10
9.	76	Male	L2 Fracture	MIS	L1-3-5	None	32	132	28:38	4:46
10.	40	Female	Spinal canal stenosis	MIS	L5-S1	Decompression and TLIF Cage L5/S1	40	297	10:31	2:37
11.	80	Female	Spinal canal stenosis	Open	Extension of fusion from Th12-L1 onto L3-5-S1	Decompression LW-S1	20	130	10:22	1:43
12.	79	Female	Th12 Fracture	MIS	Th10/11-L1/2	Secondary surgery: Corpectomy Th12, expandable cage via lateral approach	62	180	31:46	3:58

## Data Availability

The data in this study are available on request from the corresponding author. The data are not publicly available due to privacy restrictions.

## Supplementary Materials

The following are available online at https://zenodo.org/record/5634940#.YbAgHLoRVGO, Operative video (Attachment).
